# Classifying grey seal behaviour in relation to environmental variability and commercial fishing activity - a multivariate hidden Markov model

**DOI:** 10.1038/s41598-019-42109-w

**Published:** 2019-04-04

**Authors:** Floris M. van Beest, Sina Mews, Svenja Elkenkamp, Patrick Schuhmann, Dorian Tsolak, Till Wobbe, Valerio Bartolino, Francois Bastardie, Rune Dietz, Christian von Dorrien, Anders Galatius, Olle Karlsson, Bernie McConnell, Jacob Nabe-Nielsen, Morten Tange Olsen, Jonas Teilmann, Roland Langrock

**Affiliations:** 1 Marine Mammal Research, Department of Bioscience, Aarhus University, Frederiksborgvej 399, DK-4000 Roskilde, Denmark; 2 Department of Business Administration and Economics, Bielefeld University, Universitätsstraße 25, 33615 Bielefeld, Germany; 3 Department of Aquatic Resources, Swedish University of Agricultural Sciences, Lysekil, SE-45321 Sweden; 4 National Institute for Aquatic Resources, Technical University of Denmark, Kemitorvet, Kgs. Lyngby, DK-2800 Denmark; 5 Thünen Institute of Baltic Sea Fisheries, Alter Hafen Süd 2, D-18069 Rostock, Germany; 6 Department of Environmental Research and Monitoring, Swedish Museum of Natural History, Box 50007, SE-104 05 Stockholm, Sweden; 7 Sea Mammal Research Unit, University of St Andrews, St Andrews, KY16 8LB United Kingdom; 8 Evolutionary Genomics Section, Natural History Museum of Denmark, Department of Biology, University of Copenhagen, Øster Voldgade 5–7, DK-1350 Copenhagen K, Denmark

## Abstract

Classifying movement behaviour of marine predators in relation to anthropogenic activity and environmental conditions is important to guide marine conservation. We studied the relationship between grey seal (*Halichoerus grypus*) behaviour and environmental variability in the southwestern Baltic Sea where seal-fishery conflicts are increasing. We used multiple environmental covariates and proximity to active fishing nets within a multivariate hidden Markov model (HMM) to quantify changes in movement behaviour of grey seals while at sea. Dive depth, dive duration, surface duration, horizontal displacement, and turning angle were used to identify travelling, resting and foraging states. The likelihood of seals foraging increased in deeper, colder, more saline waters, which are sites with increased primary productivity and possibly prey densities. Proximity to active fishing net also had a pronounced effect on state occupancy. The probability of seals foraging was highest <5 km from active fishing nets (51%) and decreased as distance to nets increased. However, seals used sites <5 km from active fishing nets only 3% of their time at sea highlighting an important temporal dimension in seal-fishery interactions. By coupling high-resolution oceanographic, fisheries, and grey seal movement data, our study provides a scientific basis for designing management strategies that satisfy ecological and socioeconomic demands on marine ecosystems.

## Introduction

Quantifying behavioural decisions made by free-ranging animals is fundamental to understand how they use the environment, which has implications for population ecology and conservation biology^1,2^. Ecologists increasingly rely on electronic telemetry to collect movement data of individual animals and to assess behaviour indirectly^3–5^. Classifying behaviour from movement data is possible by assuming that different behavioural states are reflected by specific characteristics of individual movement paths. For example, a foraging state is often characterised by tortuous movements, while a travelling state is typically reflected by directed movements^6,7^. One particularly flexible statistical approach to classify movement patterns into different underlying behavioural states is the use of hidden Markov models (HMMs^8,9^). A basic HMM for movement time series consists of two stochastic processes: an observed movement process and an underlying (hidden) state process, where the latter can serve as a proxy of the ‘true’ behavioural process^9^. Besides quantifying state occupancy, HMMs can also be used to compute state-switching probabilities as a function of covariates, thus allowing quantification of behavioural adaptations in response to internal stimuli and external stimuli from the environment^8,9^.

Air-breathing marine mammals spend most of their active time underwater requiring telemetry and sensors to collect data on their movements. Applications of HMMs on marine mammal movement data have focused mainly on classifying behavioural states using either vertical movement (dive) parameters^10–12^ or horizontal movement parameters^13,14^. However, combining horizontal and vertical movements into one model is likely to capture behavioural states more accurately^15^. Only few marine mammal movement studies currently exist that adopt such a multivariate HMM approach^16,17^. In these cases, however, the tracking period was either limited to a few hours due to reliance on short-retention tags^16^ or the influence of environmental conditions on state-switching was not incorporated directly into the model^17^. Here, we developed a multivariate HMM based on high resolution movement data of grey seals (*Halichoerus grypus*) in the southwestern Baltic Sea by integrating both horizontal (step length, turning angle) and vertical (dive depth, dive time, post-dive duration) movement parameters collected over several months and multiple individuals. Moreover, we considered a variety of static and dynamic environmental conditions in the model to directly estimate their effect on behavioural state classification (i.e. a covariate-dependent transition HMM).

Grey seals were historically abundant in the Baltic Sea with a population estimate of ca. 100 000 individuals in the late 19^th^ century^18^. However, the population was drastically reduced, first by bounty hunting to mitigate conflicts with commercial fisheries, and later by reduced fecundity due to organochlorine pollutants such as PCB and DDT^18–20^. Protection of species and drastic reductions of toxic substances released into the system has led to the gradual recovery of several top predator populations in the Baltic Sea including grey seals^18,19^. Indeed, grey seals are now recolonizing the southwestern Baltic Sea where they only occurred sporadically since 1900^21^. Yet, with the recovery of grey seals, former conflicts with commercial fisheries are resurfacing. The most common aspects underlying the conflict between grey seals and fisheries are *i*) large scale competition between seals and fishermen for particular fish stocks, *ii*) the direct damage of fish caught in nets or longlines, *iii*) damage to fishing gear when seals remove fish from fishing nets and *iv*) bycatch, where seals get incidentally caught in fishing gear^21,22^. Throughout the Baltic Sea, all four aspects have been documented^20,23,24^ but direct removal of fish by grey seals from fishing nets and damage to fishing gear is the primary reason currently fuelling discussions in the southwestern Baltic Sea about renewed grey seal population control through culling^21^. Although grey seal movement and diving patterns have been studied in some detail throughout their European^25,26^ and North American^27,28^ range, variation in movement behaviour of grey seals in relation to environmental conditions and commercial fishing activity is poorly documented. Lack of updated information on the spatial and temporal interactions between seals and coastal fisheries is especially problematic as it aggravates the debate on the seal-fishery conflict that is not based on scientific evidence. Indeed, such ecological knowledge is a prerequisite to guide resource management strategies, and to facilitate the design of potential mitigation measures.

We provide the first detailed analysis of the movement behaviour of grey seals living at the southwestern frontier of their Baltic distribution as a function of environmental variability and commercial fishing activity. We considered static (e.g. bathymetry, sediment type and slope) and dynamic (e.g. sea surface temperature and salinity) environmental variables known to influence marine mammal movement behaviour^29,30^ and fitted these directly into a multivariate HMM to assess variation in movement behaviour while at sea. To quantify movement behaviour of grey seals near commercial fishing operations, we included proximity to active fishing net locations in real time as a dynamic covariate in the analysis.

## Methods

### Study area and grey seal biology

**Figure 1:**
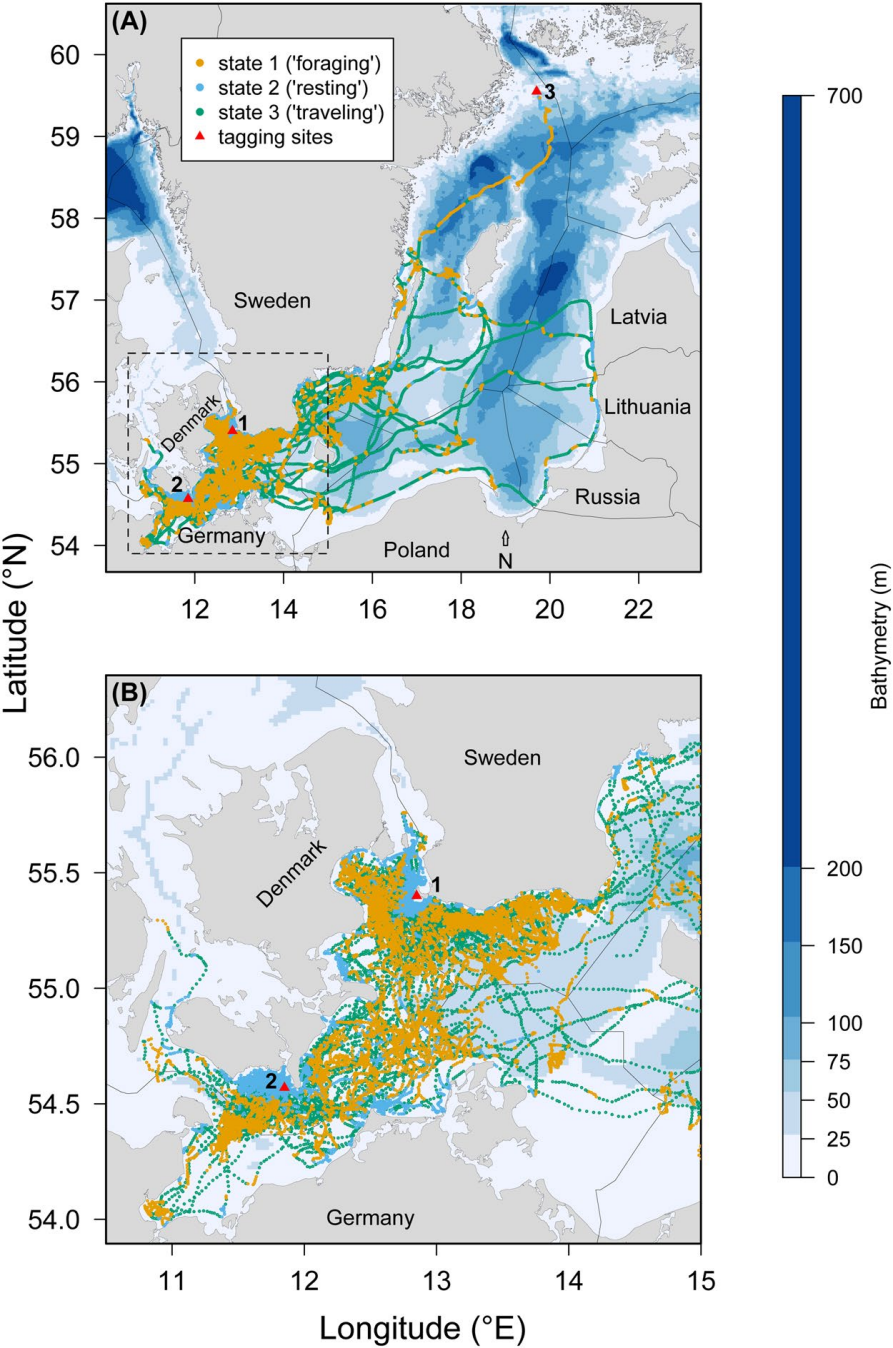
Map of the study area showing (**A**) all the location data of grey seals (N = 11), collected between 2009 and 2013, color-coded by the estimated behavioural state and the three tagging sites (1 = Måkläppen, 2 = Rødsand, 3 = Svenska stenarna). (**B**) Close-up of the grey seal locations and behavioural states as indicated by the dashed square in (**A**).

The study area covers the southwestern part of the Baltic Sea (Fig. 1). Most of the study area has shallow water depths (<60 m) but depths down to 200 m do occur. The sediment types found in the area are clay (40%), mud (21%), sand (20%), hard bottom complex (16%) and bedrock (3%). The Baltic Sea has variable water temperatures and is the largest brackish sea in the world with a sea surface salinity gradient ranging from ca 25–30 Practical Salinity Unit (PSU) in the Belt Sea to about 1 to 2 PSU in the northeast^31,32^. Vertical temperature and salinity gradients with thermoclines and haloclines as well as hypoxia in deeper waters are present throughout the area.

Baltic grey seals give birth to pups in February and March, an adaptation to breeding on ice, although parts of the population also breed on land^33^. Baltic grey seals moult their fur in May and June, during which time the greatest proportion of the population is hauled out on land^34^. Grey seals are considered opportunistic feeders, and within the Baltic, diet varies between regions indicating that prey availability plays a large part in diet composition^35^. In the southern Baltic, preferred grey seal prey items are cod (*Gadus morhua*), black goby (*Gobius niger*), round goby (*Neogobius melanostomus*), plaice (*Pleuronectes platessa*), herring (*Clupea haerengus*) and sprat (*Sprattus sprattus*). Young Baltic grey seals (<2 years of age) have been recorded to eat smaller prey and more non-commercial fish species than older seals (e.g., viviparous eelpout (*Zoarces viviparus*) and sand eels (*Ammodytes* spp.)), while older juveniles have diets that are similar to adult grey seals^36^.

### Collection of movement data

A total of 11 grey seals (eight males and three females) were captured and tagged at the haul-out sites on Rødsand, Denmark (N = 5 juveniles; fall 2009 and 2010), Måkläppen, Sweden (N = 5 juveniles; fall 2012) and Svenska Stenarna, Sweden (N = 1 juvenile; spring 2012) (Table 1, Fig. 1). Seals tagged at Rødsand and Måkläppen were caught in series of floating monofilament entanglement nets (180 mm stretched mesh, 4.5 m deep and 70 in length) and brought to shore for handling. Seals were caught in the nets for a maximum of eight hours during night time before being extracted at first day light. The seal tagged at Svenska Stenarna was caught in a hoop-net directly on the haul-out site. For each seal, the sex, weight, and standard length were recorded, and age was estimated based on size (Table 1). All individuals were judged to be in good condition and equipped with a GPS/GSM tag (Global Positioning System/Global Systems for Mobile Communications^37^) containing a pressure sensor that was attached with epoxy glue dorsally on the neck. The pressure sensor recorded three characteristics of each individual dive including max depth (m), total dive time (s) and post-dive surface time (s). The tag is essentially a data logger that uses Fastloc GPS to try to acquire a location during surfacing events. Movement data are recorded and stored onboard continuously^38^. When the tag comes within mobile phone (GSM) coverage, the stored data (locations and dive metrics) are transmitted to a server ashore. Internal data storage was sometimes exceeded before the tag was within GSM coverage to offload the data. This produced occasional gaps in the movement data, with a total maximum loss of movement data of one entire day for some individuals. In addition, movement data collected the first 24 hours after tagging were discarded, which is common practice in marine mammal movement studies to ensure that behavioural bias following capture is removed from the data as much as possible^39^. Handling time for each individual (weighing, measuring and tagging, including waiting time for the epoxy glue to harden) was approximately 30 minutes after which the seals were released back into the water at the same location where they were captured.

**Table 1:** Overview of sex, weight, standard length, age class, tagging location, tracking duration and number of dives recorded for the 11 grey seals used in this study.

ID no	Sex	Weight (kg)	Std. length (cm)	Age class	Tagging location	Tracking start	Tracking end	Tracking days	No of dives
GS01	F	48	112	juvenile	Rødsand	25-Oct-2009	6-Feb-2010	104	27055
GS02	M	44	117	juvenile	Rødsand	1-Nov-2009	12-Apr-2010	162	33291
GS03	M	43	119	juvenile	Rødsand	7-Oct-2010	29-Mar-2011	173	17778
GS04	M	42	115	juvenile	Rødsand	8-Oct-2010	23-Feb-2011	138	27888
GS05	M	40	122	juvenile	Rødsand	9-Oct-2010	6-Apr-2011	179	26114
GS06	F	41	—	juvenile	Svenska stenarna	27-Mar-2012	20-Aug-2012	146	50879
GS07	M	60	138	juvenile	Måkläppen	14-Nov-2012	28-Jan-2013	75	20546
GS08	M	66	128	juvenile	Måkläppen	15-Nov-2012	26-Feb-2013	103	29589
GS09	F	63	115	juvenile	Måkläppen	7-Dec-2012	19-Feb-2013	74	24112
GS10	M	56	121	juvenile	Måkläppen	7-Dec-2012	6-Mar-2013	89	29952
GS11	M	79	150	juvenile	Måkläppen	8-Dec-2012	19-Feb-2013	73	16691

Standard length for GS06 was not recorded. Movement data collected the first 24 hours after tagging were discarded to limit potential capture/tagging-related effects on movement behaviour in the data.

### Processing of movement data

Location data were restricted to movements made at sea (Fig. 1), as the multivariate HMM was designed to incorporate both horizontal and vertical (dive) movement data (as described in the Statistical analysis section). A total of 303 895 dives were collected over 1 316 tracking days with a mean (SD) number of dives of 27 627 (9 294) across individuals (N = 11) and a mean (SD) of 247 (79) dives per tracking day per individual. Raw data screening revealed bouts of similar movements where for any observation the dive characteristics (e.g. maximum depth) and horizontal displacement were nearly identical to several previous and subsequent dives. Preliminary fitting of the multivariate HMM with the raw data (dive-by-dive scale) led to numerical problems in the maximum likelihood estimation and extreme residual autocorrelation. To address these issues, we divided the movement data into dive sequences (i.e. batches) containing 10 dives each to avoid numerical problems and to reduce serial correlation. Thus, for the final analysis we considered the following behavioural metrics operating on the batch level:

- maximum depth (m) averaged over the 10 dives in the batch;

- dive duration (s) averaged over the 10 dives in the batch;

- surface duration (s) averaged over the 10 dives in the batch;

- horizontal step length (m) calculated from the start of the first until the end of the tenth dive in the batch;

- turning angle (radians) as the angle between compass directions of the displacement in the previous and the current batch.

### Environmental data

The covariates considered in the analysis reflect the main environmental characteristics of the study area, which were also expected to influence seal movement behaviour. Initially we considered five static environmental variables (Supplementary Fig. S1): bathymetry (m), seabed slope (°) and distance (km) to coast (the Euclidian distance to closest land mass including mainland or islands), which were calculated based on a digital terrain model (300 m resolution) of the region. The fourth static environmental condition considered was distance (km) to nearest haul-out site, which was calculated as the Euclidian distance between each seal location and the closest known haul-out site in the region. Tagged seals used multiple haul-out sites in the region (Supplementary Fig. S1), which were identified based on GPS locations that were acquired on land or sandbanks without dive information. The final static variable considered was sea floor sediment type, which was a categorical variable including sand, clay, mud, bedrock and hard bottom complex. Sediment type was extracted from a raster file (300 m resolution) accessed through the Baltic Sea Management – Nature Conservation and Sustainable Development of the Ecosystem through Spatial Planning project (BALANCE: http://www.helcom.fi/baltic-sea-trends). Due to extremely high collinearity between distance to coast, distance to haul-out site, and bathymetry (VIF > 25; *r* > 0.85), we retained only bathymetry for further analyses as it was the variable least correlated to the other covariates considered and known to be related to grey seal movement in the Baltic^40^.

We considered two dynamic environmental covariates (Supplementary Figs S2–S3) including sea surface temperature (°C) and sea surface salinity (PSU). Data on sea surface temperature and salinity (top 1 m of water column) were hourly raster data (2 km resolution) accessible from the Copernicus Marine Environmental Monitoring Service (CMEMS: http://marine.copernicus.eu/). CMEMS raster data were model prediction values as derived by the Forecasting Assimilation Baltic Sea Margin model^41^. We appended the raster value of sea surface temperature and salinity for each hour to the seal data using GPS locations and the associated time stamp. Besides the environmental variables, we also considered sex (male/female) of the seal as an intrinsic variable in the analysis.

### Commercial fisheries data

Data on commercial gill net fishing activity were collected for Danish, German and Swedish vessels operating within the study area and for the entire grey seal tracking period. Although a variety of fish species is targeted in the regional fishing industry^42^, cod is the species with the highest contribution in weight to catch ratio followed by flounder (*Platichthys* spp.) and herring. We focused on gillnets only as comparable data on use of fykes, fish traps and hooks were not available. Both large (≥12 m) and small (<12 m) gillnet vessels operated in the study area. Small gillnet vessels in Denmark and Germany are not required to track and report their offshore route and data on fishing locations are therefore lacking. In contrast, small Swedish gillnet vessels are monitored through GPS and fishing locations and soak time (start/end time of nets in waters) are reported by the fishermen in logbooks. Hence, we collected logbook data of Swedish vessels operating within the study area and period to obtain gillnet fishing locations and their associated soak time. Large vessels (≥12 m) are required to track and report their movements with a Vessel Monitoring System (VMS). We collected VMS data of all large Danish, German and Swedish vessels operating within the study area and during the study period. To obtain gillnet fishing locations and the start time of when the net was placed in the water, we processed the VMS data using the approach detailed in Bastardie *et al*.^43^. In brief, this approach aims to detect fishing events and locations for all VMS-equipped vessels (one position every 1 h interval) by analysing variation in the speed profile during a trip at sea. Based on speed thresholds, the vessels trajectory is classified into periods of drifting, fishing and steaming events. The lower threshold was 0.5 knots while the upper speed threshold was specific to each vessel and detected automatically. Most gillnet fishing events occurred between 0.5 and 3 knots in vessel speed. For each fishing event a time stamp is then extracted from the VMS data indicating when a net was placed into the water (Supplementary Fig. S4). For Swedish vessels logbook data also provided an end time for when the nets were taken out (i.e. soak time). A time stamp for when the net was taken out of the water was missing for Danish and German data. As such, we assumed soak time for all Danish and German fishing events detected during a trip to be similar to the mean soak time as derived from all Swedish vessels combined (Fig. Supplementary Fig. S5), which was 0.9 days. Through the above VMS and logbook data processing procedure, we compiled a database with a total of 24 018 gill net fishing locations between October 2009–March 2013; the period that overlapped spatially and temporally with the seal movement data (Supplementary Fig. S6). Swedish fishing locations constituted 71% of the data, Danish fishing locations constituted 22% of the data, while German fishing locations constituted 7% of the data. Screening of logbook data on fishing operations did not reveal the use of light or bait. Although the use of deterrents such as pingers (underwater devices that emit noise to deter harbour porpoises (*Phocoena phocoena*) away from gill nets) are mandatory according to EU council regulation in most of the study area^44^, deterrents are not used widely and systematically. After the fishing location and soak time database was compiled, we identified for each seal location all gillnet fishing locations that were active (i.e. soaking) by overlaying the date-time stamp in the seal movement data with the soak time in the fishing location dataset. We then calculated the Euclidian distance (km) between each seal location and the nearest known location of an active fishing net.

### Statistical analysis

**Figure 2:**
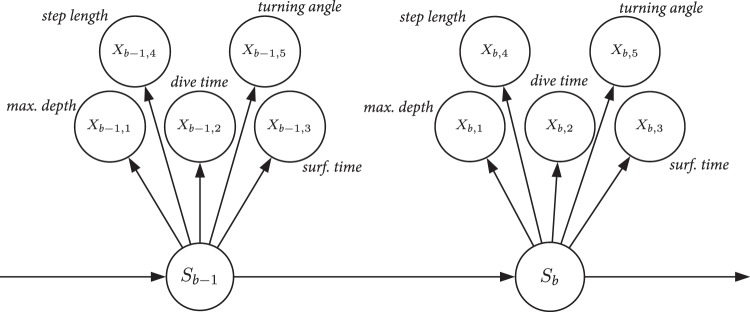
Dependence structure of the multivariate HMM fitted to the grey seal movement data. *X*_b,1_ is the average maximum depth within dive batch *b*, *X*_b,2_ is the average dive duration within dive batch *b*, *X*_b,3_ is the average post-dive surface duration within dive batch *b*, *X*_b,4_ is the step length over dive batch *b*, and *X*_b,5_ is the turning angle associated with dive batch *b*.

We developed a multivariate HMM to analyze the grey seal movement data. In the model, the distribution of the observations (**X**_*b*_) made in movement batch *b* is one out of *N* possible distributions as determined by an underlying or “hidden” state process *S*_*b*_ (Fig. 2). Our model was multivariate as **X**_*b*_ is a vector comprising maximum depth, dive duration, surface duration, horizontal step length and turning angle as averaged over the batch of dives. We used an unobserved first-order N-state Markov chain, which assumes that the probability of being in the current state is determined only by the previous state^9,10^. More specifically, the way the states evolve over time is completely specified by the one-step state transition probabilities denoted as, for *i*, *j* = 1, …, *N*. Moreover, the model was built under two conditional independence assumptions. First, the state active during movement batch *b* completely determines the distribution of **X**_*b*_ and second, that the observations made during the movement batch (maximum depth, X_*b*1_, dive duration, X_*b*2_, surface duration, X_*b*3_, step length, X_*b*4_, and turning angle, X_*b*5_) are conditionally independent of each other. Under the given assumptions, our model is able to capture some but not all of the complex dependence structures in the behavioural time series data whilst maintaining computational tractability, which is a recurrent issue when analysing large datasets with these types of models^16^.

The assumption of contemporaneous conditional independence allowed us to choose one class of univariate distributions for each of the five movement variables within a batch. Maximum depth, dive duration, surface duration and step length were modelled as a gamma distribution, as these were continuous positive values, where we used a parameterization in terms of the mean *μ* and standard deviation *σ*. Turning angles were modelled using a von Mises distribution, which is a circular analogue of the normal distribution.

Because the main aim of our analysis was to investigate the state-switching process of grey seal movement behaviour while at sea in relation to environmental variability, we designed the HMM to detect and classify three dominant states, namely foraging (State 1), resting (State 2) and travelling (State 3). These three states are considered the most common behavioural states within daily activity budgets of grey seals and other air-breathing marine mammals^11,45,46^. We made the state transitions dependent on the covariates by including these in the (batch-specific) state transition probabilities as follows:

$${\gamma }_{ijb}=\frac{\exp ({\beta }_{ij0}+{\beta }_{ij1}{z}_{b1}+\mathrm{..}+{\beta }_{ijp}{z}_{b1})}{1+{\sum }_{l\ne i}\exp ({\beta }_{il0}+{\beta }_{il1}{z}_{b1}+\ldots +{\beta }_{ilp}{z}_{b1})}$$

Here *z*_*b*1_, …., *z*_*bp*_ are the values of *p* covariates observed at the beginning of movement batch *b*. The specification used here is that of multinomial logit modelling for categorical regression, but where the categories are the different states the process may switch into (i.e. covariate-dependent state transitioning^47^). Forward selection based on information criteria (AIC and BIC) was used to assess the influence of the seven covariates considered. To improve numerical stability of the parameter estimation, each covariate was standardised to have zero mean and unit standard deviation. Similar to the problems regarding selection of the number of states, the AIC is known to be overly generous when it comes to including covariates^48^, while the BIC will be overly conservative in our case given that the observations are not independent of each other (and hence the penalty will be too large). Therefore, we did not select one best model but rather used both information criteria to evaluate and present “an envelope” of reasonable models (a concept similar to the model confidence set^49^. For each of the covariates, we computed the probabilities of occupying the different states as a function of the covariate value - in each case fixing the values of the other covariates at their respective means^47,50^.

We used the forward algorithm to evaluate the likelihood of the HMM described above. The forward algorithm exploits the dependence structure of the HMM to calculate the likelihood recursively i.e. step-by-step traversing along the time series, updating the likelihood as well as the probability of being in the different states in every step^9^. The relatively low computational cost of evaluating the likelihood via the forward algorithm renders it analytically feasible to estimate the model parameters by numerically maximizing the likelihood^51^, which we achieved using the optimization routine “nlm” in R^52^. Finally, to estimate the most likely sequence of behavioural states in the data as derived by the covariate-dependent HMM, we applied the Viterbi algorithm^9^ to assign a state to each observation. Total computation time to fit the multivariate HMM was eight hours on an Intel i7–2600 desktop with 3.4 GHz and 12 GB RAM.

To assess diel variation in behaviour, we computed state-activity budgets for each hour while at sea and for each individual separately. To do so, we calculated for each hour of the day the frequency that a seal was classified to be in one of the three states (based on Viterbi).

## Results

### State-allocation and goodness-of-model-fit

**Figure 3:**
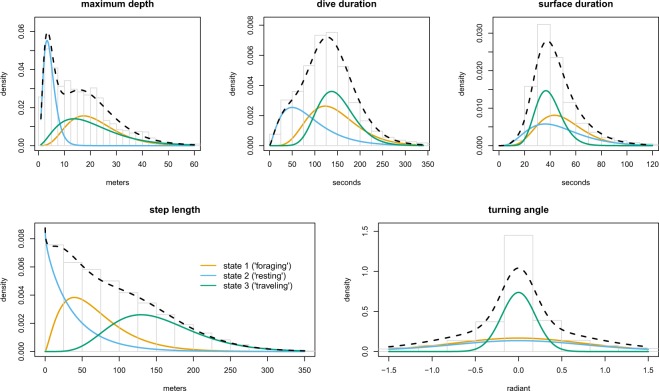
Histograms of the movement variables maximum depth, dive duration, surface duration, step length, and turning angle, respectively, overlaid with the state-dependent distributions as estimated for these variables by the HMM. The state-dependent distributions were weighted according to the proportion of time spent in the different states, as inferred using Viterbi, and the dashed lines indicate the associated marginal observation distributions under the full model.

**Figure 4:**
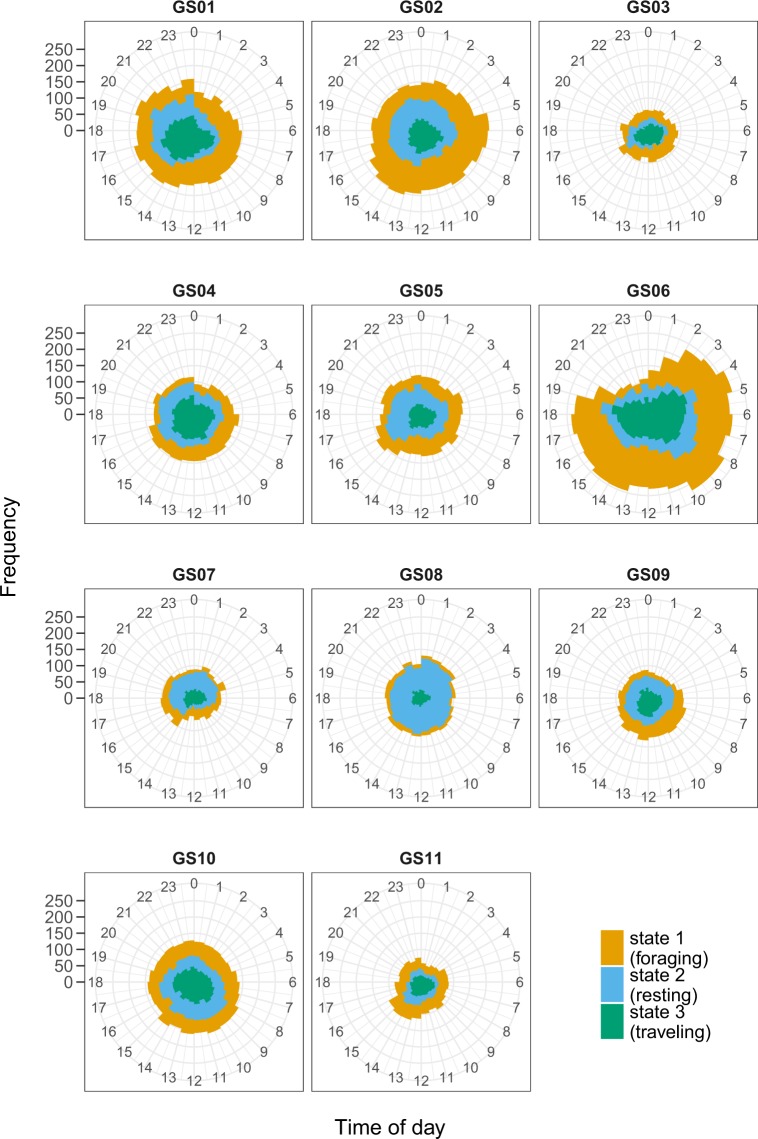
State-time budgets for each individual grey seal showing for each hour of the day the frequency of being in one of the three behavioural states.

The estimated state-dependent distributions for the five movement variables (maximum depth, dive duration, surface duration, step length, and turning angle) are provided in Fig. 3. States 1 and 3 were similar in terms of duration and depth of dives, but differed substantially in horizontal movement characteristics. State 3 included large step lengths and was associated with turning angles centred around 0 (i.e. directed movement), while state 1 included intermediate step lengths and a wide range in turning angles (undirected movement). We interpreted these distributions for state 1 and state 3 as proxies for ‘foraging’ and ‘travelling’ behaviour respectively. State 2 included the shortest and shallowest dives and was associated with rather short step lengths and a wide range in turning angles (i.e. undirected movement), which we interpreted and defined as ‘resting’ behaviour while at sea. Diel state-activity budgets for each individual, after excluding the part of the tracks where seals were on land, are provided in Fig. 4. All seals were found to occupy each behavioural state each hour of the day although there was a tendency for increased frequency of foraging behaviour during the day (05:00 to 15:00) and increased frequency of resting behaviour at night (16:00 to 04:00). No clear pattern in diel variation of travelling behaviour was found (Fig. 4). When calculating the proportion of time spent in the three behavioural states over the total tracking period of each individual seal (Table 1; Fig. 4), the average proportion of time spent in the resting state was 0.27 (range: 0.17–0.49), the average proportion of time spent in the foraging state was 0.35 (range: 0.24–0.42) and the average proportion of time spent in the travelling state was 0.37 (range: 0.21–0.49).

The marginal distributions of the five movement variables as estimated by the HMM also corresponded well with the underlying empirical distributions (Fig. 3). However, inspection of model pseudo-residuals revealed that the structure in some of the variables’ marginal distributions were not fully captured (Supplementary Fig. S7), and residual autocorrelation remained in all variables, despite the batch processing procedure (Supplementary Fig. S8). Nonetheless, for the step length variable, which is the main determinant of the classification into the three behavioural states (as evidenced by the higher overlap of the other variables’ state-dependent distributions Fig. 3), the goodness of fit of the model pseudo-residuals was satisfactory (Supplementary Fig. S7).

### State occupancy in relation to environmental variability

**Figure 5:**
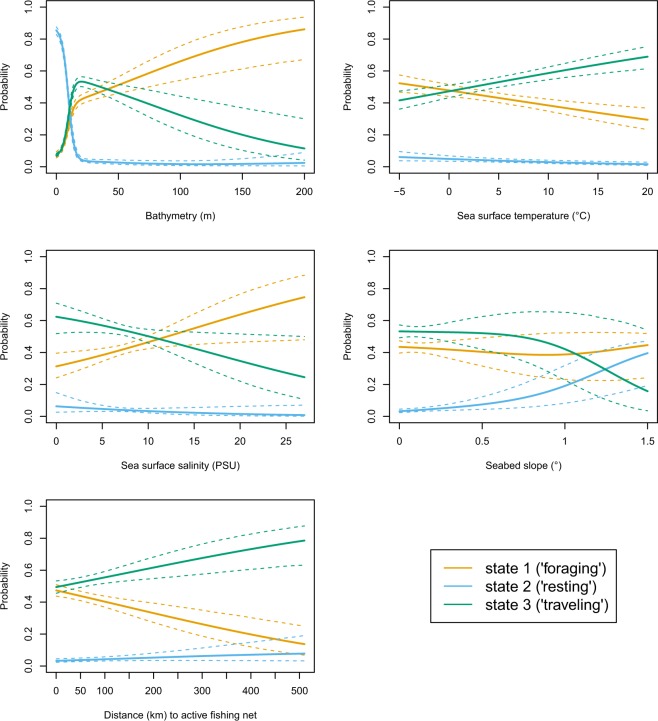
Probabilities (mean and 95% CI) of occupying the three behavioural states as a function of the covariates included in the multivariate HMM. Probabilities were calculated for each covariate and state separately by fixing the values of the remaining covariates at their respective means. All estimates were made using the categorical covariates sex (male) and sediment type (sand) as reference categories. CIs for the probabilities were obtained based on Monte Carlo simulation from the estimators’ approximate distribution as implied by maximum likelihood theory. Coefficients of the multinomial logistic regression underlying this figure are provided in Supplementary Table S1.

Forward selection of covariates into the multivariate HMM produced different best models depending on the information criteria used (AIC or BIC). Starting with the covariate-free model, the BIC-based selection procedure led to the inclusion of bathymetry, seabed slope, and sea surface temperature (Table 2). In contrast, the AIC-based selection procedure led to the inclusion of all covariates (the full model, Table 3). Given the different complexity penalties, we expected AIC and BIC to provide substantially different results. We regard the two models chosen, by BIC and by AIC respectively, as an envelope of reasonable models. Therefore, we report patterns derived from the full AIC model so as to provide an overview of all covariate effects, even those that were deemed less relevant by BIC alone. The output of the multinomial logistic regression for the full model (beta coefficients and the 95% CI) used to predict the probability of grey seal state occupancy in relation to all covariates (Fig. 5) are provided in Supplementary Table S1.

**Table 2:** Output of forward variable selection procedure based on the information criteria BIC showing the log-likelihood, log 𝓛, BIC and ∆BIC values for the baseline model without any covariates and for models with increasingly many covariates included.

Model	Log-likelihood	BIC	∆BIC
Baseline	− 626423.4	1253221.7	1324
+bathymetry	−625756.9	1251951.1	53.4
+seabed slope	−625699.0	1251897.8	0.1
+sea surface temperature	−625667.7	**1251897**.**7**	0
+sex	−625639.4	1251903.6	5.9

The best model is indicated in bold.

**Table 3:** Output of variable selection procedure based on the information criteria AIC showing the log-likelihood, AIC and ∆AIC values for the full model (indicated in bold), with all covariates, and for the models that included all but one covariate.

Model	Log-likelihood	AIC	∆AIC
Full model	−625489.4	**1251170**.**8**	0
- bathymetry	−625944.8	1252069.6	898.8
- seabed slope	−625541.0	1251262.0	91.2
- sex	−625521.9	1251223.8	53
- sea surface salinity	−625496.6	1251173.2	2.4
- sea surface temperature	−625519.0	1251218.0	47.2
- distance to active fishing net	−625514.0	1251208.0	37.2
- sediment type	−625603.6	1251351.2	180.4

Bathymetry was found to be an important covariate in the model selection process, irrespective of the information criteria used (∆BIC = 53.4, Table 2 and ∆AIC = 898.9, Table 3). The probability of seals occupying the resting state was highest in areas with shallow water depth (1–20 m depths likely close to haul-out sites) and it rapidly declined as bathymetry increased (Fig. 5). The opposite trend was found for the probability of seals occupying the foraging state which was lowest in areas with shallow water depths (1–20 m depths) and increased as bathymetry increased up to a maximum depth of 200 m (Fig. 5). The probability of seals occupying the travelling state was also lowest in areas with shallow water, but peaked at around 20 m water depth, and steadily decreased thereafter as bathymetry increased (Fig. 5).

As the slope of the sea bed increased, the probability of seals occupying the travelling state decreased while the probability of seals occupying the resting state increased (Fig. 5). The probability of seals occupying the foraging state was not related to changes sea bed slope.

Sea surface temperature and salinity did not appear to influence the probability of seals occupying the resting state, yet both of these dynamic environmental conditions were related to the probability of seals occupying the foraging and travelling states, though in opposite directions (Fig. 5). The probability of seals occupying the travelling state was positively related to sea surface temperature but negatively to sea surface salinity. In contrast, the probability of seals occupying the foraging state was negatively related to sea surface temperature and positively to sea surface salinity (Fig. 5).

The categorical covariate sex appeared an important covariate in the model (∆AIC = 53, Table 3). Although the change in probability of state occupancy in relation to environmental gradients did not differ between male and female seals, differences in the intercept of the probability of state occupancy were present. As an example, the probability of male seals occupying the travelling state at sea surface salinity of 1 PSU (the intercept) was 0.6 (Fig. 5) while for females this was 0.45 (Supplementary Fig. S9). The probability of occupying the travelling state steadily declined with increasing salinity for both male and female seals in a similar fashion.

The categorical covariate sediment type was also important (∆AIC = 180.4, Table 3). This pattern was largely driven by movement behaviour of seals at sites with sediment type bedrock, a relatively rare sediment type covering 3% of the study area, where state occupancy and state-switching deviated strongly from patterns observed in the other sediment types (Supplementary Fig. S10).

### State occupancy in relation to commercial fishing activity

**Figure 6:**
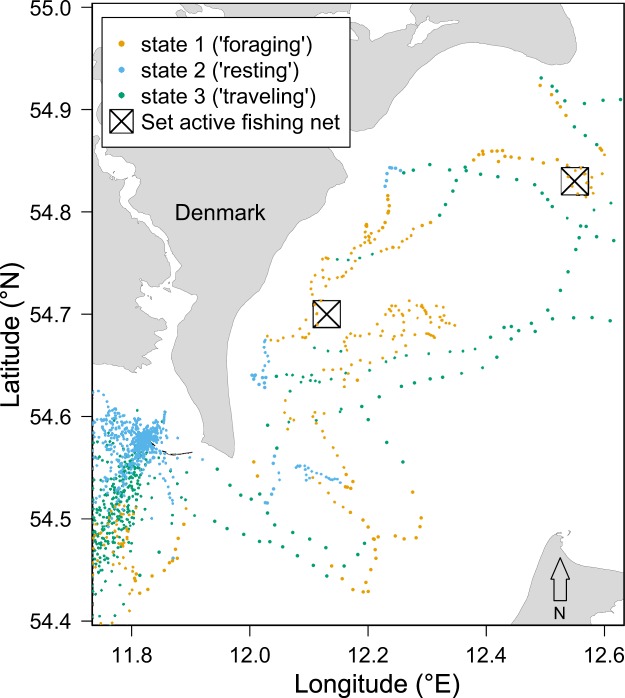
Movement track of a single grey seal for one week in the southwestern part of the Baltic showing for each location the predicted behavioural state using the Viterbi algorithm relative to the location of two active fishing nets (black squares). The fishing nets were placed in the water by a Danish fishing vessel. Seal locations in proximity to the set active fishing nets (black squares) were collected with a GPS/GSM tag between 11:30 and 14:00.

We found support for a correlation between the covariate distance to active fishing net and grey seal state occupancy (∆AIC = 37.2, Table 3). The probability of seals occupying the foraging state was highest relatively close to active fishing nets (up to < 5 km from the seal location) and steadily decreased as the distance to active fishing nets increased (Figs 5 and 6). Here, we did not observe a difference between male and female seals as the probability of occupying the foraging state in sites <5 km from active fishing nets was ca. 0.5 for both sexes (Fig. 5 and Supplementary Fig. S9). The probability of seals occupying the travelling state was equally high as the probability of being in the foraging state when active fishing nets were <5 km from the seal location but the probability of seals occupying the travelling state increased with distance to the active fishing nets. The probability of seals occupying the resting state was only weakly positively correlated with distance to active fishing nets (Fig. 5). Overall, 3.3% of the seals’ time at sea was spent <5 km from a known active fishing net, increasing to 5.9% of their time <10 km from nets, and 9.5% of their time <15 km from active fishing nets. Of the movements <5 km from an active fishing net, seals were in the foraging state 51% of the time, in the travelling state 42% of the time and in the resting state for the remaining 7% of the time. Movements that were <10 km and <15 km from active fishing nets were generally distributed equally between the three different states (ca 33% of the time).

## Discussion

### Movement behaviour in relation to environmental variability

Based on a combination of high-resolution oceanographic and movement data, analysed within a multivariate HMM framework, we found that the likelihood of seals occupying the foraging state was greatest in deeper, colder and more saline waters. The combined relationship of sea surface salinity, temperature, and bathymetry may suggest that seals target foraging sites with greater mixing of nutrients and oxygen through the water column^53^. In addition, these environmental conditions (salinity, temperature, nutrients, productivity) generally increase in a gradient from northeast to southwest in the Baltic Sea^54^, and are important determinants of the distribution and diversity of the marine fish community in this system^55^. Hence, the environmental conditions used here can be considered good proxies of spatiotemporal changes in marine fish abundance and distribution. The salinity gradient is particularly important in this respect as more saline areas in e.g. southwestern Baltic and the inner Danish waters have higher marine fish species richness and biomass than the brackish inner Baltic Sea^56,57^. As such, this finding is in line with the recent notion that the west-ward recolonization of grey seals into Danish waters could, at least partly, be due to individuals tracking prey availability into more saline waters^21^.

The need to consider multiple temporal scales, static and dynamic environmental conditions to explain grey seal movement behaviour has been highlighted also in other parts of their distribution. For example, the behavioural classification and state-activity budget of grey seals in the north-western part of the North Sea was tightly linked to seasonal variation in habitat conditions^46^. Because of a bias towards movement data collected during winter and spring in our study, we were unable to accurately assess seasonal variation in behaviour but we do show that state-activity budget of grey seals varies among individuals and over the diel timescale (Fig. 4). Indeed, grey seals were typically allocating more time to foraging behaviour during daylight hours and more time to resting behaviour during night time. More generally, grey seals spent 17%−49% of their time resting, 24%−42% foraging and 21%−49% travelling. Surprisingly, these state-activity estimates are similar to those reported for northern (*Callorhinus ursinus*) and Antarctic fur seals (*Arctocephalus gazella*)^58,59^ despite these species living in completely different habitats.

Distance to haul-out site is also known to impact models of grey seal movement behaviour^25,60,61^. Seals frequently use haul-out sites to care for new born pups, to moult, and to rest on land, though seals also rest extensively while at sea^46^ as this study also highlights. However, due to collinearity issues, we did not use distance to haul-out site in our analysis and instead focused on bathymetry. This was because the latter is a key environmental condition in grey seal space use patterns within their Baltic Sea distribution^40^ and also because the tagged seals used multiple haul-out sites throughout the area and tracking period. We do not argue that distance to haul-out site is unrelated to grey seal movement behaviour, but instead that the use of this variable is most relevant when modelling the dynamics of foraging trips made by individuals that travel larger distances over longer time periods to and from the same haul-out site (i.e. central-place foraging behaviour)^25,60,61^ compared to the grey seals in the south-western Baltic, a relatively landlocked system.

### Movement behaviour in relation to commercial fishing activity

Seal foraging and travelling behaviour was clearly related to proximity to active gill net fishing locations. This finding provides further indirect evidence that the environmental variables considered here capture at least some of the variation in fish abundance in this system as commercial fishing operations are expected to target sites where fish abundance is high so as to maximize catch per unit effort. We found that the likelihood of grey seals occupying a foraging state at locations with active fishing nets was relatively high (ca. 50%) for both males and females. Our model results are, however, correlative and should not be interpreted as causation. Hence, we do not postulate that grey seals in this region necessarily switch to feeding behaviour because there is a fishing net. Instead, a more likely explanation is that grey seals move to their preferred feeding areas (here identified as deeper, colder and more saline waters) to search for prey, which are areas also used by the fishing industry. Indeed, spatial overlap between seals and commercial fishing operations has often been studied and used as a measure to identify potential hotspots for seal-fisheries conflict^62–64^. Our study, however, also provides a measure of temporal overlap between seal foraging behaviour and fishing activity. By linking grey seal locations to the nearest known active fishing net in near real-time, we found that the tracked seals spent 3.3% of their time at sea in sites <5 km from an active fishing net. Although this is likely a minimum estimate, due to incomplete data on fishing activity, the generality of this degree of overlap is unknown as similar measures from other areas are lacking. Nonetheless, this value could be interpreted as rather low temporal interaction between grey seal foraging activity and local fishing operations. It is important to note that our sample of tracked seals did not contain any adult males, which is a segment of the population believed to have the highest direct interaction with fishing gear in the Baltic Sea^36,65,66^, which contrasts with observations in other regions of the world^67,68^. Future tracking studies should therefore focus on a more balanced sample of individuals across the entire age-spectrum of the population. Moreover, the gillnet database was incomplete (lacking accurate information on soak time from German vessels as well as gill net locations from Danish and German fishing vessels <12 m in size and from other Baltic countries) and contained only locations of where gill nets were released into the water. However, nets are typically >100 m long, and it is therefore possible that some seal locations were closer to an active fishing net than we were able to compute. Although our dataset on fishing activity is arguably one of the most extensive and detailed thus far, at least for the Baltic Sea, a more complete dataset including fishing locations and soak times of all vessels (as in Sweden) and countries would reduce uncertainty in the results. However, collating such a dataset would likely require a change in legislation where recordings of logbook and/or VMS data on all commercial vessels is a requirement. In spite of these limitations, we suggest that the temporal dynamics are an essential but often overlooked dimension in the seal-fishery conflict debate that should be considered in more detail in the future when identifying potential conflict areas.

Our modelling framework can easily be applied to other regions and marine (mammal) species to quantify spatial and temporal overlap between human activity and animal behaviour as long as similar movement and environmental data are available. A natural extension of our approach to further address the seal-fisheries conflicts would be to test the impact of potential mitigation measures and the consequences at the population-level and landscape-scale. For example, acoustic alarms (i.e. seal scarers) that emit very loud sound pulses could in principle be attached to fishing vessels or gillnets in an attempt to spatially deter seals away from fishing nets. This management strategy could reduce the direct removal of fish by seals from fishing nets and associated damage to fishing gear but also reduce the chances of bycatch. However, seal scarers may have large effects on other marine life like harbour porpoises^69^ and the successfulness of acoustic alarms in reducing direct removal of fish by seals from gillnets has yet to be fully evaluated. Yet, other sounds (pingers) have been shown to successfully reduce harbour porpoise bycatch, and when combined with other mitigation measures it also had a positive impact at the population-level^70^.

### Future prospects

The application of HMMs and other behaviour classification models to animal movement data is rapidly growing^7^. The state process in most HMMs, including ours, should be considered a proxy for the underlying sequence of behavioural states, though caution is needed not to over-interpret the completely data-driven meaning of the states^11^. This is especially a concern when the number of behavioural states selected from the data is arbitrarily large and within an unsupervised framework (i.e. lack of validation data on behavioural states). We limited our classification to the three most common behavioural states of grey seals, although seals, as most species, likely use a much wider and more complex suite of behaviours during their lifetime. Clearly, there is potential for more precise state estimation of animal movement data, especially when adopting a supervised classification approach that is guided and tested by empirical observations e.g. through the use of animal borne video recorders^71^. Moreover, video footage can provide valuable information on spatial and temporal variation in diet composition facilitating the classification of more foraging types. Collecting high-resolution environmental data by the tag directly (e.g. via temperature and salinity sensors^72^) is also possible now, reducing the reliance on courser remote-sensing and modelled environmental data. When coupled with accelerometer tags^73^ movement ecologists can estimate the behaviour of wild animals and their interactions with the environment in unprecedented detail^15^. The increasingly fine-scale resolution and amount of movement data collected nowadays also present challenges in terms of data handling and statistical analysis^7,74^.

Fortunately, analytical procedures are continuously being extended to address the issues that follow with the high frequency movement data being collected^11,75,76^. We foresee great value in linking behavioural-state classification models such as ours with dynamic energy budget models and spatially explicit agent-based simulation models^77^. Such an approach opens up new ways to study population dynamics and the impacts of anthropogenic and environmental stressors through bottom-up movement processes^70,78^. Ultimately, movement-based spatially explicit population models will facilitate the design and testing of mitigation efforts aimed at e.g. alleviating seal-fisheries conflict but also of broader management and conservation strategies to balance ecological as well as socioeconomic demands on marine ecosystems successfully.

### Ethics Statement

All applicable international, national and/or institutional guidelines for the care and use of animals were followed. Activities related to seal tagging at Rødsand, Denmark, were carried out with permission of the Environmental Protection Agency (Ministry of Environment and Food of Denmark, permit number SNS-342-00042) and the Animal Experiments Inspectorate (Ministry of Environment and Food of Denmark, permit number: 2010/561-1801). Activities related to seal tagging at Måkläppan, and Svenska Stenarna, Sweden, were carried out with permission of the Swedish Environmental Protection Agency (permit number: NV-04536-15), the Central Animal Ethics Committee (permit number: A-52-15) and the Country Board of Skåne (permit number: 521-18979-2012).

## Supplementary information

Supporting Information
