# Supplementary material for: Classifying grey seal behaviour in relation to environmental variability and commercial fishing activity - a multivariate hidden Markov model
Source: Sci Rep. 2019 Apr 4;9:5642. doi: 10.1038/s41598-019-42109-w (PMC6449369; doi:10.1038/s41598-019-42109-w)
Supplement: Supplementary file 1 — Supporting Information [file 41598_2019_42109_MOESM1_ESM.docx]

**Supplementary Information**

**Classifying grey seal behaviour in relation to environmental variability and commercial fishing activity - a multivariate hidden Markov model**

Floris M. van Beest^1^**^*^**, Sina Mews^2^, Svenja Elkenkamp^2^, Patrick Schuhmann^2^, Dorian Tsolak^2^, Till Wobbe^2^, Valerio Bartolino^3^, Francois Bastardie^4^, Rune Dietz^1^, Christian von Dorrien^5^, Anders Galatius^1^, Olle Karlsson^6^, Bernie McConnell^7^, Jacob Nabe-Nielsen^1^, Morten Tange Olsen^8^, Jonas Teilmann^1^, Roland Langrock^2^

*^1^ Marine Mammal Research, Department of Bioscience, Aarhus University, Frederiksborgvej 399, DK-4000 Roskilde, Denmark*

^2^ *Department of Business Administration and Economics, Bielefeld University, Universitätsstraße 25, 33615 Bielefeld, Germany*

*^3^ Department of Aquatic Resources, Swedish University of Agricultural Sciences, Lysekil SE-45321, Sweden*

*^4^ National Institute for Aquatic Resources, Technical University of Denmark, Kemitorvet, Kgs. Lyngby DK-2800, Denmark*

*^5^ Thünen Institute of Baltic Sea Fisheries, Alter Hafen Süd 2, D-18069 Rostock, Germany*

*^6^ Department of Environmental Research and Monitoring, Swedish Museum of Natural History, Box 50007, SE-104 05 Stockholm Sweden*

*^7^ Sea Mammal Research Unit, University of St Andrews, St Andrews, KY16 8LB, United Kingdom*

*^8^ Evolutionary Genomics Section, Natural History Museum of Denmark, Department of Biology, University of Copenhagen, Øster Voldgade 5-7, DK-1350 Copenhagen K, Denmark*

*Author for correspondence ([flbe@bios.au.dk](mailto:flbe@bios.au.dk)).


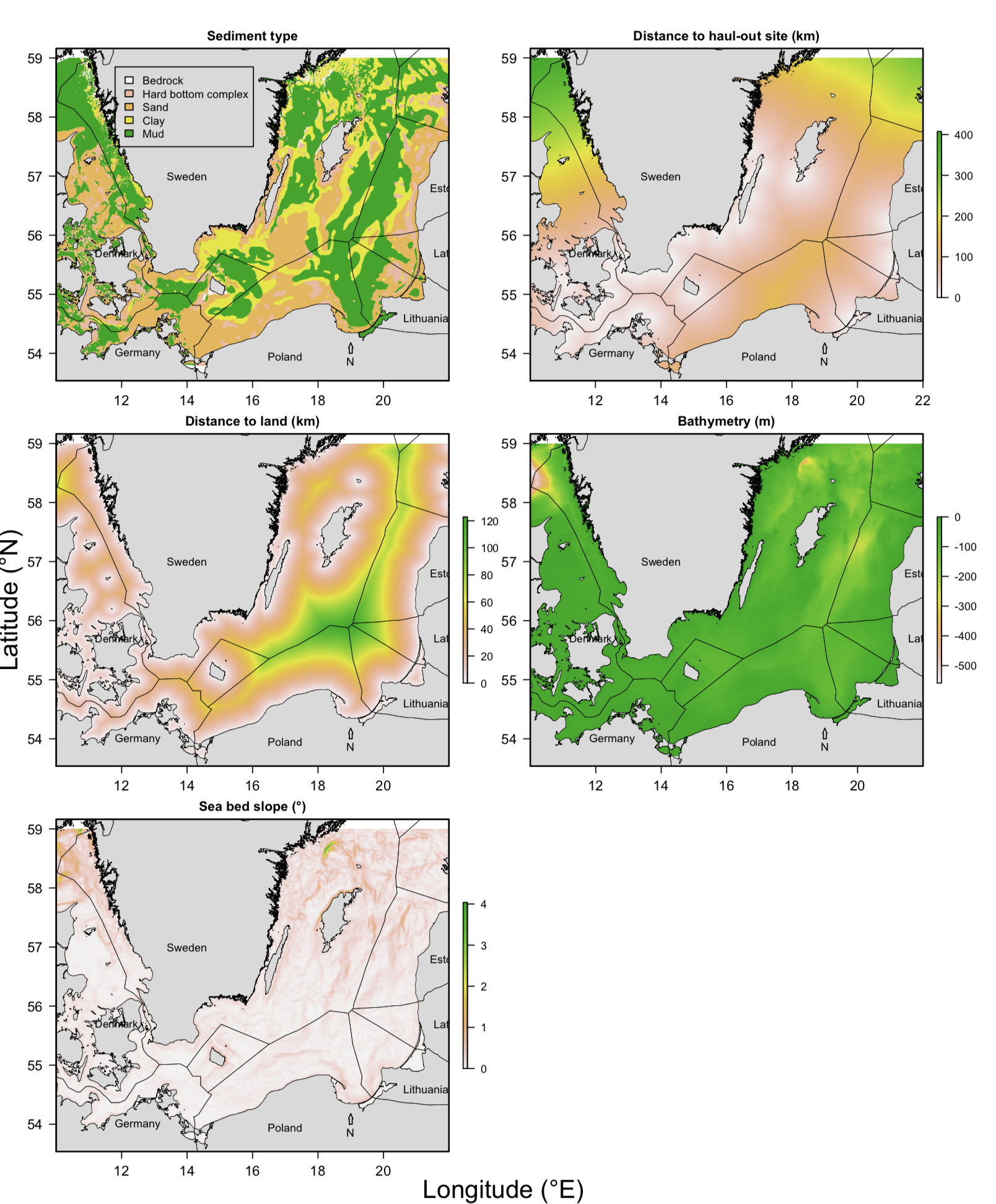


**Figure S1**: Maps of the static environmental conditions considered as potential covariates in the HMM.


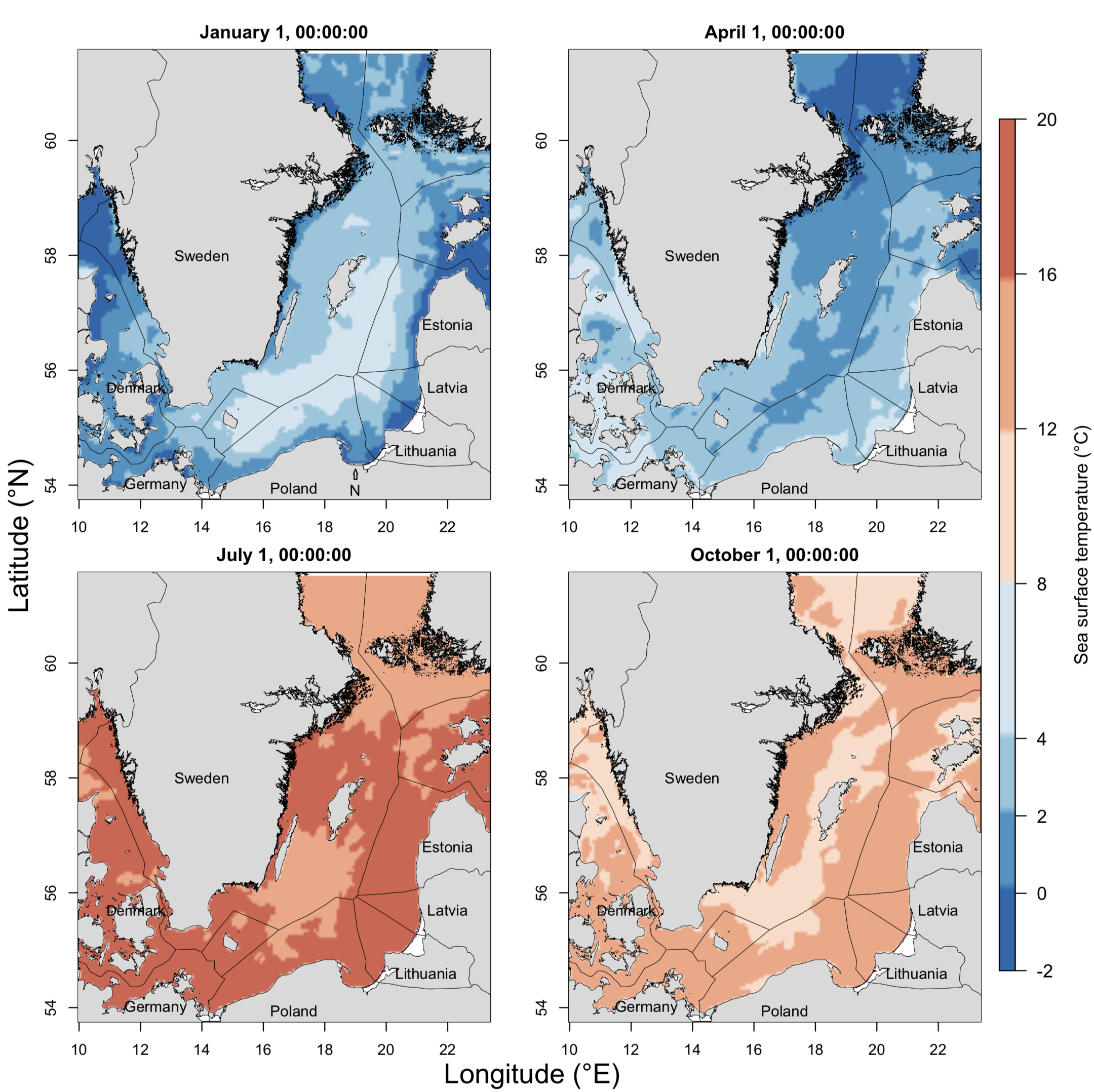


**Figure S2**: Example of raster maps of sea surface temperature (°C) during midnight on the first day of January, April, July and October. Temperature data were available on an hourly scale for the complete study period (2009-2013).

**
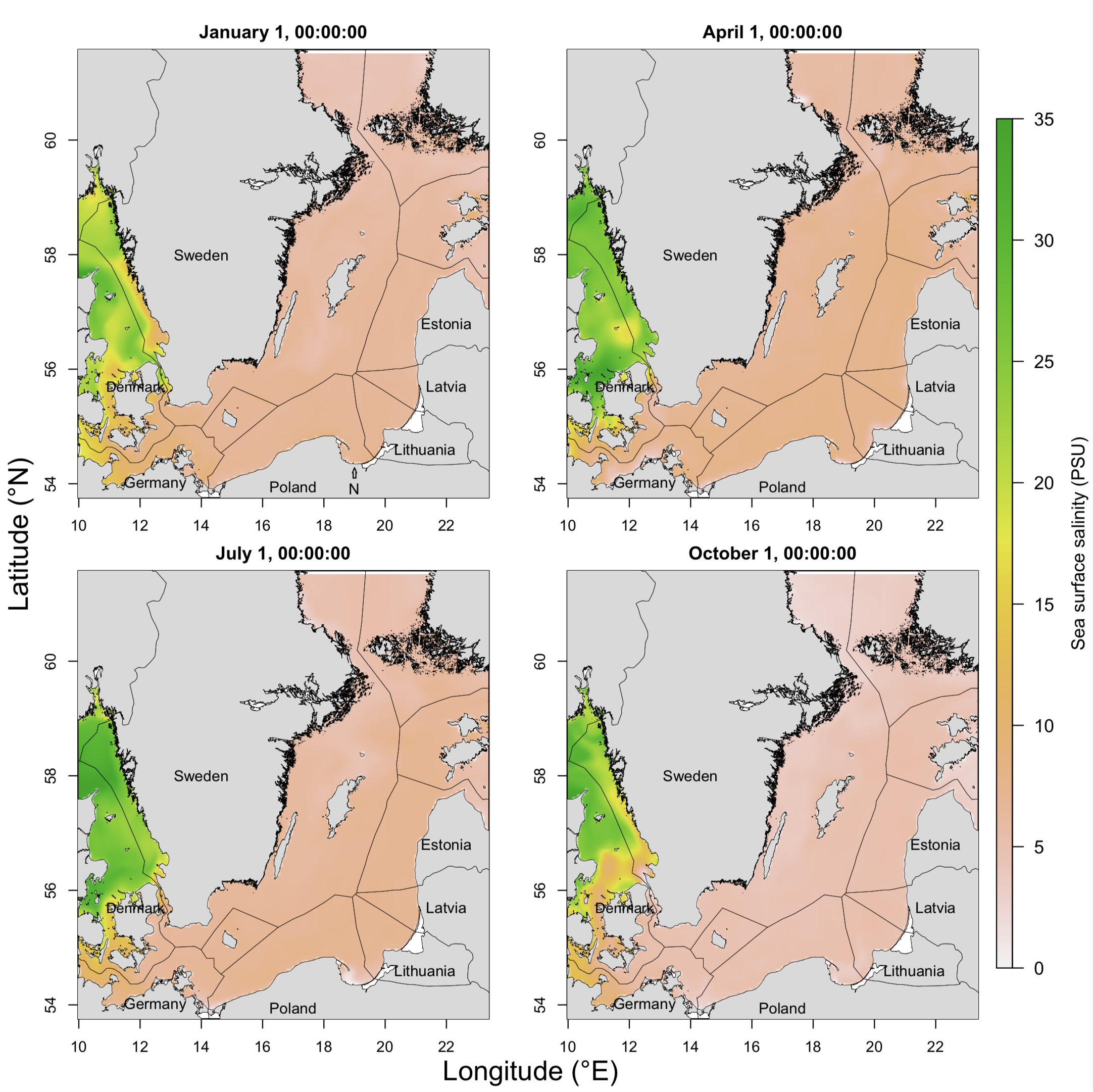
**

**Figure S3**: Example of raster maps of sea surface salinity (PSU) during midnight on the first day of January, April, July and October. Salinity data were available on an hourly scale for the complete study period (2009-2013).

**Figure S4**: Circular histograms showing for each study year and country the hour of the day that a gillnet was set in the water, which was estimated by analyzing variation in the speed profile of all VMS-equipped vessels (one position every 1h interval) during a trip at sea. See main article for more detail on the processing procedure of commercial fisheries data. Swedish fishing locations constituted 71% of the data, Danish fishing locations constituted 22% of the data while German fishing locations constituted 7% of the data.


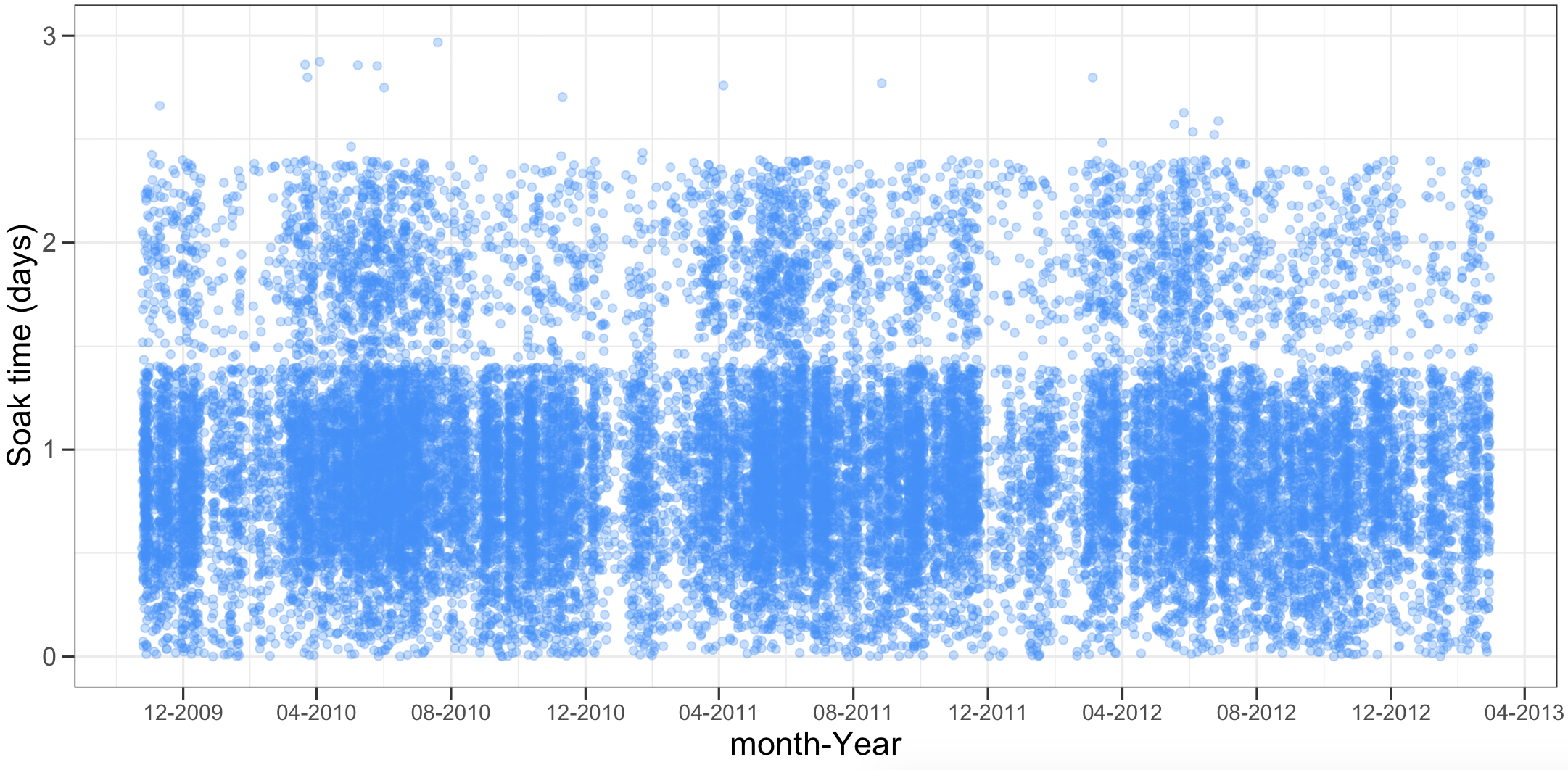


**Figure S5**: Soak time (days) of fishing events for all Swedish (both large ≥12m and small <12m vessels) gillnet vessels. Soak time could not be calculated for Danish and German fishing events and were set to 0.9 days, which was the mean soak time as derived from all Swedish data combined.


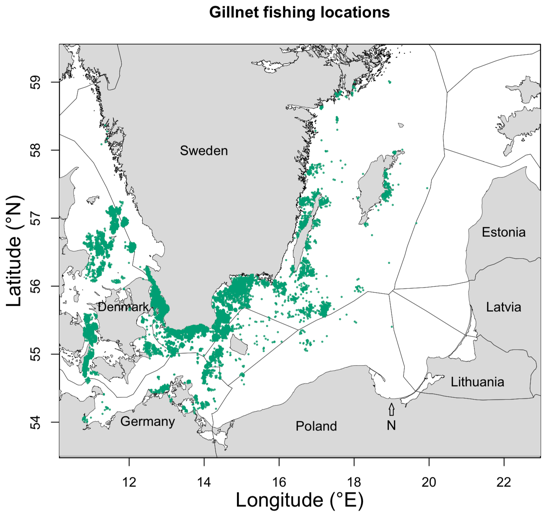


**Figure S6**: Map showing estimated gill net fishing locations (N=24018) in the study area during the study period (2009-2013, see Table 1 in main article). For each fishing location of Swedish and Danish vessels date-time stamps were available for when the net was placed in the water and for when the net was taken out of the water (i.e. used to calculate soak time). Soak time for fishing events by German vessels could not be calculated and was set to the mean soak time of Danish and Swedish vessels combined (mean soak time of 0.9 days). Swedish fishing locations constituted 71% of the data, Danish fishing locations constituted 22% of the data while German fishing locations constituted 7% of the data. Date-time stamps and locations were used to match the seal - and gillnet fishing locations to calculate the distance of seals to the nearest known active gillnet in real time. Note that shown locations are approximate as a small amount of noise was added to each coordinate for confidentiality purposes.


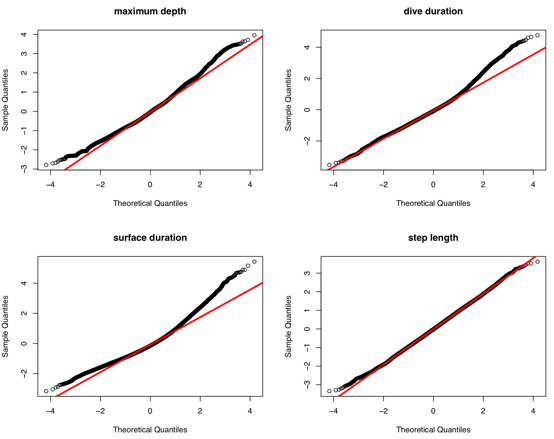


**Figure S7**: Quantile-quantile plots of the pseudo-residuals for the movement variables included in the three-state multivariate HMM fitted to the grey seal movement data.

**Figure S8**: Plot of the autocorrelation functions of the pseudo-residuals for the movement variables included in the three-state multivariate HMM fitted to the grey seal movement data.

**Table S1**: Coefficients (Beta, lower and upper bound of 95% CI) of the multinomial logistic regression model results used to predict the probability of grey seal state occupancy as a function of seven covariates (Fig. 4 in the main article).

| Parameters | State 1 → State 2 | | | State 1 → State 3 | | | State 2 → State 1 | | | State 2 → State 3 | | | State 3 → State 1 | | | State 3 → State 2 | | |
| --- | --- | --- | --- | --- | --- | --- | --- | --- | --- | --- | --- | --- | --- | --- | --- | --- | --- | --- |
|  | Beta | lower | upper | Beta | lower | upper | Beta | lower | upper | Beta | lower | upper | Beta | lower | upper | Beta | lower | upper |
| Intercept | -4.16 | -4.44 | -3.87 | -2.18 | -2.57 | -1.8 | -3.35 | -3.44 | -3.27 | -2.64 | -2.74 | -2.54 | -2.16 | -2.64 | -1.69 | -7.1 | -7.33 | -6.86 |
| Sex* |  |  |  |  |  |  |  |  |  |  |  |  |  |  |  |  |  |  |
| Female | 0.09 | -0.03 | 0.22 | -0.58 | -0.7 | -0.45 | -0.25 | -0.4 | -0.1 | -0.94 | -1.03 | -0.85 | -0.35 | -0.6 | -0.09 | -0.51 | -1.56 | 0.54 |
| Dist. fishing net | 0.22 | -0.12 | 0.57 | -0.02 | -0.12 | 0.07 | -0.14 | -0.37 | 0.1 | 0.06 | -0.03 | 0.16 | -0.18 | -0.41 | 0.05 | 0.21 | -0.47 | 0.89 |
| Sea temperature | 0.08 | -0.14 | 0.31 | 0.1 | -0.08 | 0.29 | 0.52 | 0.42 | 0.62 | 0.15 | -0.02 | 0.31 | -0.12 | -0.42 | 0.18 | 0.18 | -0.05 | 0.41 |
| Sea salinity | -0.16 | -0.28 | -0.04 | -0.15 | -0.26 | -0.05 | 0.05 | -0.12 | 0.21 | 0.09 | -0.05 | 0.22 | -0.01 | -0.56 | 0.54 | 0.02 | -0.83 | 0.87 |
| Seabed slope | 0.42 | -0.44 | 1.27 | 0.02 | -0.06 | 0.11 | 0.43 | 0.34 | 0.52 | 0.18 | -0.17 | 0.53 | 0.03 | -0.25 | 0.31 | 0.23 | -1.64 | 2.09 |
| Bathymetry | 0.11 | -0.27 | 0.49 | -0.23 | -0.36 | -0.1 | 0.93 | 0.84 | 1.01 | 0.35 | 0.12 | 0.59 | 0.04 | -0.75 | 0.83 | -8.24 | -9.75 | -6.73 |
| Sediment type* |  |  |  |  |  |  |  |  |  |  |  |  |  |  |  |  |  |  |
| hard bottom | 0.76 | 0.53 | 0.99 | 0.14 | -0.02 | 0.3 | 0.28 | 0.17 | 0.38 | 0.85 | 0.76 | 0.94 | 0.04 | -1.52 | 1.59 | -0.42 | -1.73 | 0.9 |
| clay | 0.18 | -0.31 | 0.68 | 0.87 | 0.78 | 0.95 | 0.78 | 0.66 | 0.9 | -1.09 | -2.24 | 0.06 | 0.01 | -0.25 | 0.27 | 1.2 | -0.79 | 3.18 |
| mud | -0.29 | -0.7 | 0.11 | 0.33 | 0.15 | 0.51 | 0.17 | 0.07 | 0.27 | 0.33 | -0.09 | 0.74 | 0.12 | -0.95 | 1.19 | 0.02 | -0.68 | 0.73 |
| bedrock | 0.34 | 0.14 | 0.54 | -1.63 | -1.75 | -1.51 | -1.91 | -2.03 | -1.78 | -3.17 | -3.4 | -2.94 | 1.53 | 0.98 | 2.08 | 0.49 | -0.47 | 1.46 |

* Reference category is male [Sex] and sand [Sediment type]

**Figure S9**: Equilibrium probabilities (mean and 95% CI) of occupying the three behavioral states for female seals in sediment type sand as a function of the covariates included in the multivariate HMM. Equilibrium probabilities were calculated for each covariate and state separately by fixing the values of the remaining covariates at their respective means.

**Figure S10**: Equilibrium probabilities (mean and 95% CI) of occupying the three behavioral states for males in sediment type bedrock as a function of the covariates included in the multivariate HMM. Probabilities were calculated for each covariate and state separately by fixing the values of the remaining covariates at their respective means.
